# Aptasensors Based on Non-Enzymatic Peroxidase Mimics: Current Progress and Challenges

**DOI:** 10.3390/bios14010001

**Published:** 2023-12-20

**Authors:** Anna S. Davydova, Mariya A. Vorobyeva

**Affiliations:** Institute of Chemical Biology and Fundamental Medicine SB RAS, Akad. Lavrentiev, 8, 630090 Novosibirsk, Russia; kuzn@niboch.nsc.ru

**Keywords:** aptamers, aptasensors, peroxidase mimics, peroxidase-like DNAzyme, nanozymes, metal-organic frameworks

## Abstract

Immunoassays based on antibodies as recognizing elements and enzymes as signal-generating modules are extensively used now in clinical lab diagnostics, food, and environmental analyses. However, the application of natural enzymes and antibodies has some drawbacks, such as relatively high manufacturing costs, thermal instability, and lot-to-lot variations that lower the reproducibility of results. Oligonucleotide aptamers are able to specifically bind their targets with high affinity and selectivity, so they represent a prospective alternative to protein antibodies for analyte recognition. Their main advantages include thermal stability and long shelf life, cost-efficient chemical synthesis, and negligible batch-to-batch variations. At the same time, a wide variety of non-protein peroxidase mimics are now available that show strong potential to replace protein enzymes. Here, we review and analyze non-protein biosensors that represent a nexus of these two concepts: aptamer-based sensors (aptasensors) with optical detection (colorimetric, luminescent, or fluorescent) based on different peroxidase mimics, such as DNAzymes, nanoparticles, or metal-organic frameworks.

## 1. Introduction

Enzyme immunoassays have established themselves as robust tools that provide the possibility of the specific and sensitive determination of certain analytes. High affinity to the analyte based on the use of antibodies is joined there with high sensitivity thanks to signal amplification in enzymatic reactions. In particular, ELISA now represents one of the most popular lab diagnostic methods applied in clinics and hospitals around the world due to its easy operation, possibility of automatization and multiplexing, and wide spectra of biomarker analytes available [1].

Nevertheless, analytical systems where antibodies as recognizing elements meet natural enzymes as signal generators possess a number of drawbacks that originate from the protein nature of both of these elements. We should mention here their relatively high manufacturing costs, thermal instability that requires cold chain transport and refrigerated storage, and especially lot-to-lot variations brought by a biotechnological way of production [2]. The problems of insufficient validation of antibodies and their high production costs have been widely discussed in recent years, as well as the necessity of standardizing recombinant binding reagents by their sequence information to address the ‘reproducibility crisis’ [3,4]. Therefore, there is still a lot of space to create improved versions of this powerful and popular method. For instance, longitude clinical investigations in biomedicine need test systems for optical assays, particularly those compatible with formats traditional for clinical lab diagnostics, with better characteristics such as lower manufacturing costs, higher reproducibility, and sensitivity.

Oligonucleotide aptamers represent a very promising alternative to protein antibodies. Having similar characteristics in terms of binding affinity and specificity, aptamers enjoy the unique advantages of highly reproducible chemical synthesis that provide stable properties independently of a particular lot or supplier, a precisely known nucleotide sequence, and a wide range of storage and transportation conditions [5]. Moreover, numerous chemical modifications have been developed to increase the stability of DNA and RNA aptamers in biological, environmental, or food samples [6].

On the other side, a great diversity of non-protein enzyme mimics has been developed in the last two decades, demonstrating high and stable catalytic activity, ease of synthesis, and lower cost, together with sensitivity comparable to natural enzymes. For example, nanozymes could replace protein enzyme reporters in immunoassays in a number of ways (see, e.g., recent reviews [7,8]). Among enzyme mimics, those catalyzing the peroxidase reaction, which is widely used in immunoassays, attract particular attention.

A combination of these two concepts led to the appearance of a large diversity of analytical biosensors, with both keystone elements represented by non-protein molecules. In their tandem, an aptamer is responsible for selectivity due to precise target recognition, while the peroxidase mimic provides a generation of the analytical signal. These constructs represent a functional alternative for antibody-peroxidase systems. Meanwhile, aptamer + peroxidase mimic tandems are also attractive because of their principal compatibility with the same analytical protocols and devices that are now widely used for immunoassays (e.g., ELISA kits, lateral flow assays, electrochemical sensors, SPR devices, etc. [9]). What is their potential to replace the traditional antibody + enzyme design, and what advantages and pitfalls shall we meet? In the current review, we analyze aptamer-based sensors (aptasensors) with colorimetric, luminescent, or fluorescent signals generated by peroxidase mimics: DNAzymes, nanoparticles, or metal frameworks (MOFs). A number of comprehensive reviews have been published to date, with the main focus either on aptasensors [10,11] or on non-protein peroxidase mimics [12,13,14,15]. A very interesting and informative work on the topic was published in 2010 by A. Sassolas et al. [16]. However, to our knowledge, during the last decade, no reviews were published concerning the test systems with aptamer recognizing modules joined with protein-free peroxidase activity, while a lot of examples of such systems have already been accumulated (table in Appendix A).

## 2. Functional Mimics of Horseradish Peroxidase

### 2.1. Complex of Hemin with DNA G-Quadruplex

**Figure 1:**
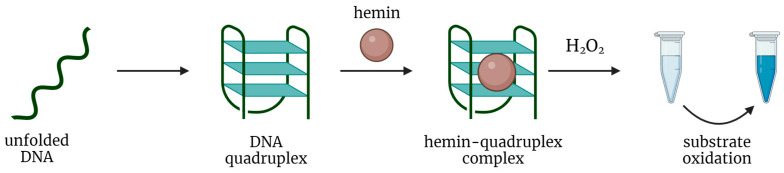
A general scheme of peroxidase-like oxidation by a hemin-DNAzyme complex.

In general, G-rich DNA sequences are able to fold into specific G-quadruplex (GQ) structures and non-covalently bind hemin. The resulting DNA-hemin complex can catalyze the oxidation of chromogenic substrates by H_2_O_2_ (Figure 1). Specifically, the oxygen atoms of guanine and hemin’s Fe(III) are brought close together, causing the hemin core to interact with G-quartets through the π-π interactions and enhancing its intrinsic ability to catalyze an oxidation reaction between hydrogen peroxide and a chromophore. It is interesting to note that hemin cofactor participates also in the catalysis of the same reaction by the natural protein, horseradish peroxidase [17]. 

As a rule, peroxidase mimics are applied in the analytical assays with the same reducing substrates (chromophores) as the natural peroxidase: 3,3′,5,5′-tetramethylbenzidine (TMB) and 2,2’-azino-bis (3-ethylbenzothiazoline-6-sulfonic acid) (ABTS) for colorimetric analysis, and luminol for chemiluminescent detection.

Such peroxidase-like complexes found their application for colorimetric signal generation in various biosensors. A combination of an aptamer and a DNAzyme seems to be especially attractive, since both molecules possess precisely the same chemical nature of nucleic acids. The constructs joining catalytic and aptameric oligonucleotides are often called ‘aptazymes’ [18].

C. Yang et al. proposed an aptasensor for the detection of a food-contaminating mycotoxin, ochratoxin A, in food [19], with one chimeric DNA molecule containing the DNAzyme and aptamer sequences. A short blocking tail was added to the 3′-end of the aptasensor to inhibit the catalytic activity of DNAzyme in the absence of an analyte. In the presence of ochratoxin A, aptamer-target binding induces the rearrangement of the whole molecule and leads to the folding of the active DNAzyme for TMB oxidation. The limit of detection (LOD) for the proposed aptasensor was 2.5 nM, which is comparable to HPLC-FD analysis. This aptasensor was used for the detection of ochratoxin A in wine samples. It should be noted that the authors developed a special protocol for processing wine samples prior to analysis with several sequential extractions of ochratoxin A by organic solvents to achieve high analytical performance. 

Teller et al. [20] applied the same strategy with a bridged AMP-binding aptamer and a DNAzyme connected via a short linker sequence at the 3′-end of the aptamer. Without AMP, DNAzyme cannot acquire an active catalytic structure and bind hemin for ABTS oxidation. In the presence of AMP, the aptamer binds its target, causing the uncaging of DNAzyme and ABTS oxidation with the generation of a colorimetric signal proportional to the AMP concentration. The LOD for the proposed aptasensor was 50 μM. The authors also designed a similar aptamer-DNAzyme hairpin for lysozyme detection, with a LOD of 0.5 pM. In both cases, hairpins were designed in such a manner that, in the absence of the target, a hairpin formation is preferable, but adding the target makes the formation of the aptamer-target complex energetically favorable. Comparison of developed aptasensors with corresponding fluorescent beacon detection showed more sensitive detection of AMP or lysozyme by hairpin aptasensors. The same research group also developed another aptasensor made of two separate DNA molecules [21], with an AMP- or lysozyme-specific aptamer linked to the DNAzyme sequence and a second blocking DNA partially complementary to the aptamer and DNAzyme sequences. In the absence of the target analyte, an aptazyme forms duplex with blocking DNA that prevents ABTS oxidation. After target addition, an aptamer dissociates from the blocking DNA and binds to its target. In turn, the DNAzyme restores its active catalytic structure with the subsequent oxidation of the ABTS substrate by H_2_O_2_. A very similar aptasensor was developed for ATP detection [22]. Aptamer and DNAzyme are joined in one DNA molecule, while the second DNA strand acts as a blocker to prevent ABTS oxidation in the absence of an analyte. The specificity of detection was proven with three other nucleotides.

**Figure 2:**
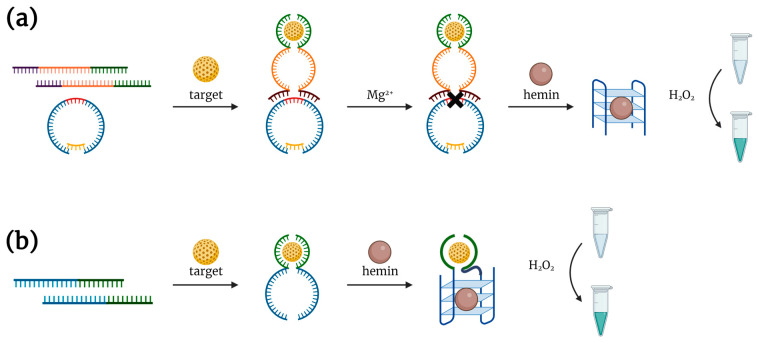
DNAzyme-based aptasensors developed in [23]. (**a**) Aptasensor consisting of quasi-circular HRP-mimicking DNAzyme with a single ribonucleobase (red color), and two separate DNAs containing partial sequences of specific aptamer and RNA-cleaving DNAzyme. (**b**) Aptasensor consisting of two separated DNAs containing partial sequences of specific aptamer and HRP-mimicking DNAzyme.

J. Elbaz et al. [23] proposed two different DNAzyme-based aptasensors for detection of low-molecular targets. The first one included a quasi-circular HRP-mimicking DNAzyme with a single ribonucleobase (rA) and two separate DNAs containing partial sequences of specific aptamer and RNA-cleaving DNAzyme (Figure 2a). In the presence of a target (ATP or cocaine), two parts of the aptamer form a complex and induce the formation of an RNA-cleaving DNAzyme. The cleavage of the rA-containing quasi-circular DNA construct gives two peroxidase DNAzyme molecules, which bind hemin and catalyze the ABTS oxidation in the presence of H_2_O_2_, providing a colorimetric readout. Notably, the working temperature of the whole aptasensor was found to be 35 °C, since at lower temperatures, an active DNAzyme structure forms even without any target, giving quite a high background signal. To simplify the detection and improve its sensitivity, the authors designed an alternative aptasensor consisting of two separated DNAs (Figure 2b). Each of them included a part of the aptamer sequence and a part of the DNAzyme sequence. In the presence of the target, the split construct assembles, giving an aptamer and a hemin-binding DNAzyme for ABTS oxidation. However, the high background signal remained the main drawback of all the proposed aptasensors due to the use of hemin that oxidizes the substrate to some extent even without a DNAzyme.

Often, G-rich aptamers contain their own quadruplex domains, thus becoming able to bind hemin and catalyze the oxidation of various substrates. For instance, target binding of a VEGF-specific aptamer induced the formation of an active GQ structure that provides luminol oxidation in the presence of hydrogen peroxide [24]. The luminescence intensity linearly increased with the rise in VEGF concentration, and the LOD was rather low (18 nM). At the same time, in the absence of a target, a significant nonspecific signal was registered due to the spontaneous GQ formation. Splitting the aptamer into two separate oligonucleotides made the GQ formation possible only in the presence of VEGF and significantly reduced the background signal. As a result, the LOD was lowered to 2.6 nM. The analogous approach was exploited for 8-Oxo-G-specific aptasensor design [25]. In the presence of an analyte, a G-rich aptamer forms a hemin-binding GQ structure and catalyzes ABTS oxidation by H_2_O_2_. The LOD for the proposed aptasensor was determined to be 140 pM. The aptasensor was used for 8-Ox-G detection in urine samples from cancer patients and a healthy donor. The sample processing prior to analysis included centrifugation and filtration through a 0.22 μm membrane. The 8-Oxo-G level was much higher in all samples from the cancer patients in comparison to a healthy donor. The obtained results were in accordance with those obtained by fluorescence analysis.

K. Mao et al. developed an aptasensor consisting of two separate DNAs. The first of them was a methamphetamine-specific aptamer, while the second represented a peroxidase-like DNAzyme split into two fragments by a sequence partially complementary to the aptamer [26]. In the absence of the target, an aptamer forms a duplex with corresponding DNA and inactivates the DNAzyme. In the presence of methamphetamine, the aptamer preferably binds to its target and releases the second oligo, thus giving the DNAzyme the possibility to form an active structure and oxidize the ABTS substrate. The LOD was 0.5 nM, much lower in comparison to an alternative detection system. The proposed aptasensor was used for methamphetamine detection in spiked urine samples filtered through a 0.22 μm syringe filter. The recovered values were in accordance with those obtained by HPLC-MS/MS. 

A similar aptasensor design was applied for the carcinoembryonic antigen (CEA) detection [27] in saliva. In the absence of CEA, the aptamer hybridizes with the central region of the split DNAzyme and blocks its catalytic activity. In the presence of CEA, aptamer binds to its target and releases DNAzyme from the duplex. Unblocked DNAzyme folds into an active structure and catalyzes ABTS oxidation. The comparison of CEA detection in spiked human saliva samples and a buffer solution showed nearly the same performance with an LOD of 1 ng/mL (5.5 pM), and the authors concluded that their assay could be applied in a salivary test. On the contrary, H. Khang et al. proposed a DNAzyme-based aptasensor for CEA detection with the optical signal inversely related to the target concentration [28]. The CEA-specific aptamer and DNAzyme were linked via a short nucleotide linker. In the absence of CEA, DNAzyme binds hemin and catalyzes the oxidation of 1,1′-oxalyldiimidazole (ODI), generating a chemiluminescent signal. The formation of the aptamer-CEA complex switches the structure of the whole aptasensor, prevents hemin-binding and substrate oxidation. The proposed aptasensor was tested using serum samples from hospital patients. The results obtained by the chemiluminescent aptasensor were in agreement with those from a commercial ELISA kit, while the LOD for the aptasensor (0.58 ng/mL) was even lower than that of ELISA.

**Figure 3:**
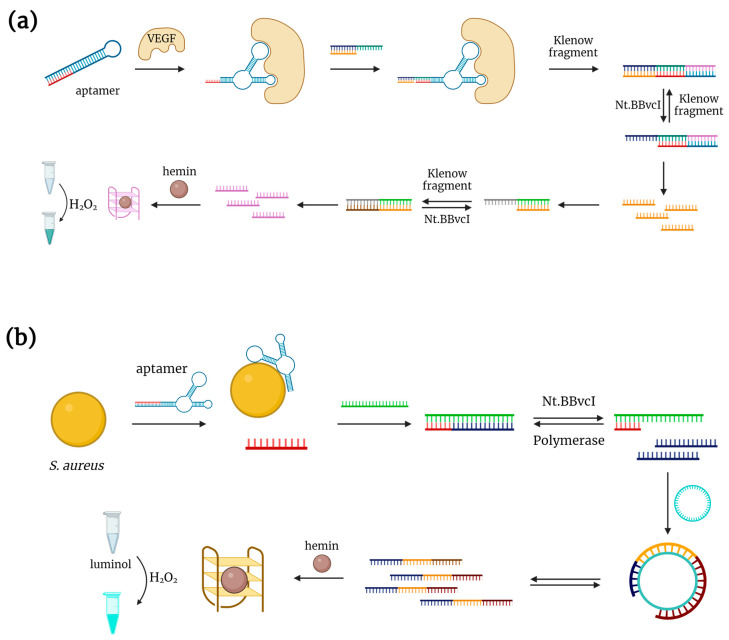
DNAzyme-based aptasensors with signal amplification. (**a**) VEGF detection system proposed by Zhang et al. [29]. (**b**) Luminescent detection of *S. aureus* developed in [30].

A peroxidase-mimicking hemin-DNAzyme complex can also serve as a signaling unit for aptasensors with signal amplification. For instance, H. Zhang et al. constructed an aptasensor for VEGF_165_ detection with the use of strand displacement amplification [29]. Initially, the aptamer is linked to an additional oligonucleotide and forms a hairpin structure. The addition of VEGF_165_ results in the rearrangement of the hairpin probe and initiates a cascade of enzymatic reactions that produce the hemin-binding GQ DNAzyme for ABTS oxidation (Figure 3a). The proposed aptasensor was tested on diluted serum samples spiked with VEGF_165_. 

Another example of an aptasensor with signal amplification was described in [30] for the detection of *Staphylococcus aureus*. Initially, the aptamer forms a duplex with a DNA oligo complementary to the 5’-end of the aptamer. In the presence of *S. aureus*, the aptamer binds to its target, thus displacing the additional DNA from the duplex. The released oligonucleotide initiates the nicking enzyme amplification reaction and the rolling circle amplification (Figure 3b). GQ DNAzyme, the main product of this cascade of reactions, binds hemin and catalyzes luminol oxidation. The LOD for this aptasensor was 5 CFU/mL. Interestingly, the developed aptasensor demonstrated an ability to discriminate between living and dead bacterial cells.

Shahsavar et al. proposed a DNAzyme-based aptasensor for ATP detection [31]. The ATP-specific aptamer was flanked by two additional sequences that form a triplex structure with DNAzyme in the absence of a target. In the presence of ATP, aptamer refolds into an active structure for analyte binding, thus disrupting the triplex with a release of DNAzyme. The latter, in turn, restores its active GQ structure, binds hemin, and catalyzes TMB oxidation. The LOD for the proposed aptasensor was 2.4 nM. The authors successfully tested it for ATP detection in spiked serum samples after filtration and 100-fold dilution.

The hemin-DNAzyme complex was successfully applied as a signal transducer within a dual-mode aptasensor for ATP detection [32]. The developed aptasensor consisted of two modules: one for direct fluorescent detection at low concentrations (down to 1 μM) and one enzymatic module for detection at high concentrations (up to 500 mM). The first module consisted of two DNAs labeled with fluorophore and quencher, acting as a typical beacon probe. The second module was linked to the ends of the first and folded into a hemin-binding quadruplex. High concentrations of ATP greatly enhanced the catalytic activity of the DNAzyme-hemin complex. Notably, AMP, ADP, and other NTPs also caused changes in fluorescent and colorimetric signals, although significantly lower than ATP. The authors also demonstrated the possibility of fine-tuning the dynamic range and the specificity of the aptasensor by varying the chemical environment, namely the Tris concentration. Moreover, the use of antisense DNAs that regulate aptamer-target binding also allowed for tuning the detection range. This dual-mode system possesses an extremely broad detection range, but the strategy of its design cannot be easily adapted for other analytes because it is based on a unique property of the ATP molecule to enhance the peroxidase activity of the hemin-DNAzyme complex. The authors conclude that the broadening of the proposed concept relies on the availability of other DNAzymes with catalytic activity controlled by diverse activators or inhibitors.

**Figure 4:**
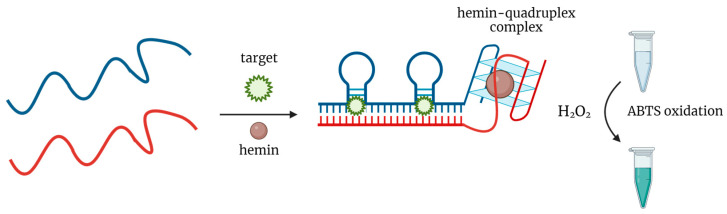
Multimodal aptasensor for small molecules detection proposed in [33].

Y. Luo et al. [33] incorporated a split hemin-binding DNAzyme into a multimodal aptasensor. The construct was made of two DNAs forming a bivalent cocaine-binding aptamer linked with a DNAzyme (Figure 4). Target binding induces appropriate folding of the aptamer, which is accompanied by the formation of an active GQ DNAzyme. A color change due to ABTS oxidation was detected by the naked eye within 5 min. The LOD for the proposed aptasensor was 1 μM. Of note, the developed aptasensor demonstrated cross-reactivity for diphenhydramine and lidocaine. The same strategy was used for the detection of the synthetic drug of abuse, methylenedioxypyrovalerone (MDPV), demonstrating the generality of the proposed aptasensor platform. This aptasensor was able to detect MDPV at 30 μM with the naked eye in 5 min, and the LOD was 3 μM. Again, the MDPV-specific aptasensor demonstrated a cross-reactivity to 11 different synthetic cathinones, so the authors suggested its potential application for the detection of the whole family of synthetic cathinones.

Y. Zhou et al. [34] combined a HRP-mimicking DNAzyme with a MUC1-specific aptamer for the detection of breast cancer exosomes. In the absence of MUC1-positive exosomes, the aptamer forms a hairpin structure that involves the sequence of DNAzyme, thus inhibiting its catalytic activity. A rearrangement of the aptamer structure after MUC1 binding disrupts the hairpin, so that the released DNAzyme can form an active hemin-binding quadruplex structure and catalyze ABTS oxidation. The LOD for the proposed system was 3.94 × 10^5^ particles/mL. The colorimetric signal generated by exosomes from tumor cells was significantly higher than that for exosomes produced by a normal cell line. Aptasensor was tested on serum samples from cancer patients and healthy donors. While the signal from the cancer group was consistently higher as compared to the healthy group, the difference was not too large, probably due to the presence of MUC1 on the surface of exosomes derived from normal cells. Nevertheless, the results were in agreement with the values obtained independently by a conventional nanoparticle tracking analysis.

L. Yang et al. selected a GQ-forming aptamer specific to the herbicide quinclorac and applied it for colorimetric detection [35]. To enhance the sensitivity of the aptasensor, the authors developed a rather sophisticated system of signal amplification based on isothermal amplification reaction. In the absence of quinclorac, an aptamer is blocked by complementary DNA. In the presence of quinclorac, the aptamer dissociates from the complex and binds its target. At the same time, the blocking DNA initiates an enzymatic cascade that results in the generation of morpholino modified DNAzyme for TMB oxidation. The color change was visible by the naked eye, and the calculated LOD was 7.1 ng/mL. The developed aptasensor was more sensitive than the immunochromatographic HPLC-UV assays. This aptasensor was used for quinclorac detection in water and soil samples. It should be mentioned that the analysis required sample pre-processing, which was quite complex and included several stages (acidification, extraction, evaporation, etc.).

Liu et al. [36] developed a system for sensitive detection of MUC-1 that combined an aptamer, DNAzyme, and magnetic nanoparticles. The aptasensor was made of two DNA molecules: the first hybrid DNA comprised MUC1-aptamer and GQ DNAzyme, and the second one, partially complementary to the aptamer, was immobilized on magnetic nanoparticles via biotin-streptavidin interactions. Without MUC1 in solution, an aptamer forms a duplex with DNA on the magnetic nanoparticles. After magnetic separation, the aptamer and DNAzyme are removed from the test tube, so no color change is observed after ABTS addition. In the presence of MUC1, the aptamer dissociates from the complementary DNA on magnetic nanoparticles to form а complex with its target. Structural rearrangement of the whole aptamer-containing molecule triggers the folding of DNAzyme and hemin binding. After the magnetic separation of nanoparticles, the hemin-DNAzyme complex remains in solution and catalyzes ABTS oxidation with color change. The calculated LOD was 5.1 nM, which is comparable to the fluorescent assay. The same results were obtained for diluted serum samples.

H. Gao et al. proposed a universal strategy for the design of a structure-switching colorimetric aptasensor with a DNAzyme-hemin complex generating an analytical signal [37]. The aptasensor consisted of two parts: the constant reporter module presented by a split hemin-binding DNAzyme and the variable recognizing module presented by a target-specific aptamer. In the absence of a specific target, DNAzyme forms a hemin-binding GQ structure, which provides ABTS oxidation with color change. In the presence of an analyte, the aptamer forms an active structure and initiates a rearrangement of the aptasensor that disrupts DNAzyme structure and decrease its peroxidase activity. The generality of the proposed strategy was proved using several aptamers characterized by different lengths, spatial structures, K_D_ and specificity. The authors developed aptasensors for the detection of Hg^2+^ ions, thrombin, sulfadimethoxine, cocaine, and 17β-estradiol.

A very interesting aptasensor for multicolor detection of ochratoxin A (OTA) was proposed in [38]. The OTA-binding aptamer was covalently linked to the GQ DNAzyme. Without OTA, the aptamer-DNAzyme chimera folds into a random spatial structure that is totally digested by Exo I nuclease. In the presence of OTA, the aptamer forms a specific complex with its target, while DNAzyme restores the active GQ structure for hemin binding and catalyzes TMB oxidation, followed by gold nanorods etching and multicolor changes. The visual LOD was 30 nM. The feasibility of the aptasensor was tested using spiked beer samples, and different concentrations of OTA provided different colors. Interestingly, in contrast to the abovementioned OTA detection in wine, which included multi-step sample processing, the proposed assay requires only the degassing of beer samples prior to the analysis.

### 2.2. Nanozymes

Various types of nanomaterials including metal nanoparticles, metal oxides, and carbon nanomaterials, can catalyze various chemical reactions, including peroxidase-like oxidation. Peroxidase-mimicking properties of platinum nanoparticles (PtNPs) were applied for an aptasensor design for thrombin detection in [39]. The authors constructed a sandwich-type assay for thrombin detection and a competitive assay for the detection of anti-thrombin antibodies. For the sandwich assay, the first biotinylated aptamer acts as a capture probe, and the second aptamer-Pt conjugate serves as a reporter. The LOD was 0.4 μM. For competitive assay, thrombin is immobilized on the plate, followed by the addition of anti-thrombin antibodies. Then, Pt-conjugated aptamers are introduced to compete with antibodies for thrombin binding. The sensitivity of the aptasensor was comparable to that of the commercial ELISA kit.

Another aptasensor recruiting a peroxidase-mimicking nanozyme was developed in [40], with Ag/Pt nanoclusters for TMB oxidation. The DNA-nanocluster conjugates were used as a reporter probe for the sandwich thrombin detection assay. The use of Ag/Pt significantly improved the sensitivity of the aptasensor as compared to the abovementioned Pt-based system; the LOD was as low as 2.6 nM.

**Figure 5:**
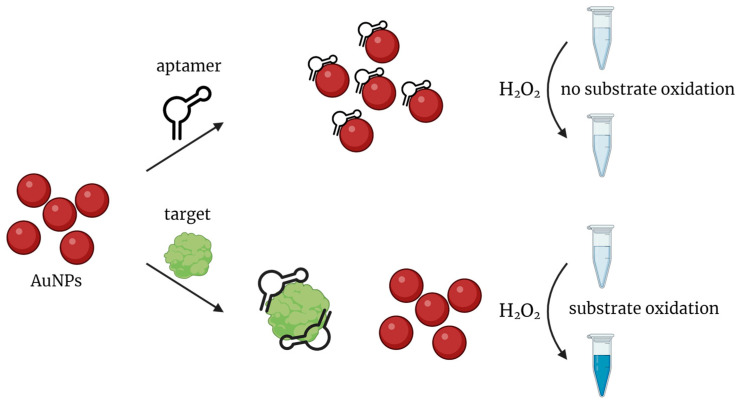
General scheme of colorimetric target detection based on peroxidase-mimicking AuNPs.

AuNPs are widely used as peroxidase-mimics for aptasensor design. In general, nonspecific absorption of nucleic acid aptamers on AuNPs inhibits their peroxidase-like activity. In the presence of a specific target molecule, the aptamer dissociates from the nanoparticle to bind the target, so AuNPs restore their catalytic properties and oxidize the chromogenic substrate (Figure 5).

For instance, a combination of AuNPs and a specific aptamer was applied for the detection of the antibiotic kanamycin [41]. In the absence of a target, an aptamer is nonspecifically bound with AuNPs, which results in the inhibition of their catalytic activity, so no color change happens. When kanamycin is present, aptamer dissociates from AuNPs to bind its molecular target, enabling AuNPs to catalyze TMB oxidation. The increase in kanamycin concentration caused an enhancement in absorbance. The aptasensor demonstrated high sensitivity (the LOD was 1.5 nM) and high selectivity in control experiments with penicillin, ampicillin, and streptomycin. The same research group developed similar aptasensors for detection of acetamiprid [42], sulfadimethoxine [43], murine norovirus [44], and *P. aeruginosa* [45]. The same detection strategy was used by Sun et al. for the detection of mycotoxin zearalenone in a buffer solution and in spiked samples of corn and corn oil [46], and also by Zhao et al. for streptomycin detection in milk [47].

Lately, an AuNPs-based aptasensor was developed for the detection of CD30-positive small extracellular vesicles (SEVs) in the blood plasma of patients with the classical form of Hodgkin lymphoma [48]. Again, while free aptamers inhibit the catalytic activity of AuNPs, in the presence of CD30-positive SEVs, aptamers dissociate from AuNPs, and nanozyme oxidizes TMB, giving a blue color. Unfortunately, the detection range in this system was quite narrow (from 5 to 40 × 10^6^ SEVs). However, the authors demonstrated statistically significant differences for plasma samples analysis between cancer patients and healthy donors.

C. Zhao et al. [49] proposed a detection system based on the inhibition of AuNPs peroxidase activity for the measurement of malachite green, an antifungal and antibiotic agent with severe toxic effects on aquatic organisms and humans. In an attempt to make the color change more prominent, they employed a cationic surfactant, cetyltrimethylammonium bromide (CTAB). Initially, positively charged CTAB interacts with citrate-capped AuNPs and induces their aggregation, inhibiting the catalytic activity. On the other side, negatively charged aptamer binds CTAB due to electrostatic interactions and enhances peroxidase-like activity of AuNPs with a dark blue color change of TMB solution. In the presence of malachite green, the aptamer forms a specific complex with its target, which leads again to the inhibition of AuNPs catalytic activity with a light blue color of solution. The LOD was 1.8 nM, which is close to those for HPLC and for the ELISA kit.

Along with ABTS and TMB, other peroxidase substrates could be exploited for aptasensor detection. For instance, positively charged ortho-phenylenediamine (oPD) was used for detection of the soluble IL-2 receptor α [50]. Without an analyte, aptamers are absorbed on the AuNP surface. Negatively charged aptamer attracts more positively charged oPD than bare AuNPs, resulting in the generation of a deep brown color solution. In the presence of the soluble IL-2 receptor α, the aptamer dissociates from AuNPs to bind its specific target, providing a decrease in the colorimetric signal. The developed aptasensor provided sensitive (the LOD was 1 nM) and selective detection of the target protein in buffer and diluted serum samples. The proposed analysis is quite fast and provided results in 25 min.

HRP-like AuNPs could also be applied as part of a sandwich detection system. J. Xie et al. [51] designed a sandwich aptasensor for C-reactive protein (CRP) detection. In this assay, a conjugate of the CRP-specific ligand citicoline with BSA was used to capture CRP in microplate wells. A CRP-specific aptamer conjugated with peroxidase-like AuNPs served as a reporter probe for protein detection. The aptasensor was used for CRP detection in blood samples of model rats with acute myocardial infarction. The linear range and LOD were comparable to those for conventional ELISA.

To improve the sensitivity of AuNPs-based aptasensors, R. Torabi et al. [52] used intercross-linked AuNPs for the detection of retinol-binding protein 4 (RBP4), an early biomarker of type II diabetes. By contrast with previous aptasensors, RBP4-specific aptamers were used for the specific capture of analyte in microplate wells. Covalent conjugates of RBP4-specific antibodies with AuNP clusters were used for analyte visualization. AuNPs were cross-linked to each other via cysteine as a linker. The resulting clusters provided a significant increase in the surface accessible for conjugating with RBP4-specific antibodies and luminol. The cross-linked AuNPs provided a 24-fold more intensive luminescent signal than traditional AuNPs. The developed aptasensor was tested on 20 serum samples from diabetes patients and healthy donors. The obtained results were consistent with those obtained by the commercial ELISA kit.

Silver nanoparticles (AgNPs) can also function as peroxidase-mimicking nanozymes. This feature was employed by P. Weerathunge [53] et al. to design an aptasensor recruiting peroxidase-like tyrosine-modified AgNPs for the detection of pesticide chlorpyrifos in spiked river water samples. As in the case of AuNPs, aptamer absorbs on the AgNPs in the absence of an analyte and inhibits their catalytic activity. In the presence of chlorpyrifos, aptamer leaves AgNPs, so they restore the activity and catalyze TMB oxidation, generating a blue color of the solution.

Another example of a nanozyme-assisted colorimetric aptasensor was developed by J. Y. Park et al. for *S. typhimurium* detection [54]. The authors employed magnetite nanoparticles and the same concept of turning on peroxidase activity in the presence of the target: aptamer binds to bacteria, and dissociates from Fe_3_O_4_ nanoparticles. The latter restore their catalytic activity and catalyze TMB oxidation with a change in solution color.

Bimetallic Au@Pd nanoparticles served as HRP-mimics for aptamer-based detection of *Campylobacter jejune* [55]. In the presence of *C. jejune*, the aptamer binds to its bacterial target, and the resulting aptamer-bacteria complexes are discarded by centrifugation. An addition of Au@Pd nanoparticles and TMB substrate leads to a color change. Without target bacteria, aptamer remains in solution after centrifugation, binds to Au@Pd nanoparticles, and inhibits their catalytic activity. This aptasensor was able to detect as few as 10 cells and distinguished *C. jejune* from *C. coli*, *H. pylori,* and *E. coli*.

Tan et al. [56] used gold core-shell nanorods GNR@Au_2_S/AuAgS/CuS as a peroxidase-mimicking nanozyme for insulin detection. The insulin-specific aptamer was covalently attached to the GNR surface. In the absence of insulin, aptamer-GNR conjugates catalyze TMB oxidation and provide the color change. Specific binding between aptamer and insulin results in the inhibition of catalytic GNR activity and a decrease in the colorimetric signal. The LOD was 0.2 pM in serum samples.

BSA-stabilized gold nanoclusters (BSA-AuNCs) possess high peroxidase-like catalytic activity and can act as a signal transducer for aptasensors. Using this possibility, Q. Chen et al. developed a BSA-AuNCs-based aptasensor for the detection of *S. typhimurium* [57]. The authors used two different aptamer-AuNC conjugates for detection of bacteria. Interestingly, the catalytic activity of AuNCs significantly increased after aptamer-target binding. The proposed aptasensor had a very low LOD (1 CFU/mL) and discriminated *S. typhimurium* from *E. coli* O157:H7, *S. aureus*, and *P. aeruginosa*.

Metal-organic frameworks, or MOFs, are a new family of peroxidase-like nanozymes that have been proposed recently for the colorimetric detection of various targets. Usually, MOFs are porous crystalline structures consisting of metal ions or clusters connected via various organic linkers. MOFs are characterized by a well-defined structure and a large surface accessible for modification and can catalyze different chemical reactions, including the oxidation of chromogenic substrates by H_2_O_2_. Y. Wang et al. proposed a MOF-based aptasensor for thrombin detection [58] based on the same principle of aptamer-induced inhibition of catalytic activity. Without thrombin, the corresponding aptamer binds to Fe-MIL-88A MOF, inhibiting its catalytic activity. After thrombin binding, an aptamer leaves MOF, resulting in TMB oxidation and color change. The LOD for the aptasensor was 0.8 nM, lower than that for other thrombin-specific aptasensors. G. K. Ali et al. designed a similar aptasensor with Cu-MOF for the detection of CRP in serum samples [59]. Apart from peroxidase-like activity, Cu-MOFs also possess fluorescent properties, so they can provide dual-mode detection. An aptamer-Cu-MOF interaction inhibits the fluorescence, and in the presence of CRP, the aptamer dissociates from Cu-MOF, restoring the catalytic activity. The LOD was very low, both in colorimetric (750 pg/mL) and fluorescent mode (40 pg/mL). An analogous dual-mode aptasensor with a femtomolar LOD was developed for thrombin detection [60].

Q. Liu et al. combined Cu-MOF with Fe_3_O_4_ nanoparticles for the magnetically controlled detection of the pesticide chlorpyrifos [61]. Aptamer was immobilized on the Fe_3_O_4_ magnetic nanoparticles, while Cu-MOF was modified by DNA complementary to aptamer. Therefore, Cu-MOF was reversibly linked with magnetic nanoparticles by complementary interactions between an aptamer and additional DNA. After magnetic separation, the whole Cu-MOF-containing composite leaves the solution and cannot catalyze TMB oxidation. The chlorpyriphos competes with DNA-Cu-MOF for aptamer-Fe_3_O_4_ binding and displaces Cu-MOF from a complex with magnetic nanoparticles. After magnetic separation, the DNA-Cu-MOF conjugate remains in the solution and provides TMB oxidation. Higher amounts of pesticide increase Cu-MOF concentration with the subsequent rise in color intensity. The obtained LOD was comparable to that of ELISA and greatly exceeded the sensitivity of electrochemical biosensors developed for the same analyte. The aptasensor was successfully validated in the analysis of spiked fruit and vegetable samples, and the results were in agreement with reference gas chromatography-MS data.

A novel soft material, metal-organic gel (MOG), combines the properties of metal-containing MOFs with a gel structure. A combination of aptamer-MOG conjugate and magnetic beads was used for the detection of mycotoxin fumonizin B1 [62]. Specific aptamer was linked to Pt NPs/Fe-MOG via biotin-streptavidin interactions. A DNA complementary to aptamer was covalently immobilized on magnetic beads, thus giving the possibility to reversibly attach a peroxidase-mimic to magnetic beads via complementary interactions between aptamer and additional DNA. After magnetic separation, MOG retains in the test tube and catalyzes TMB oxidation. In the presence of mycotoxin, the aptamer leaves the duplex and binds to its target. After magnetic separation, a supernatant containing target/aptamer/MOG complexes is discarded, making the TMB oxidation impossible. The developed aptasensor allowed for fumonizin B1 detection in samples of cornflour extracts spiked with mycotoxin. The obtained recovery results were in agreement with those obtained by a reference ELISA.

Moreover, MOF materials are compatible with each other, giving the possibility of obtaining an advanced material that combines several properties. For instance, H. Chai et al. proposed a dual-mode aptasensor for the detection of chlorpyrifos, recruiting a hybrid MOF-on-MOF nanozyme for signal generation [63]. A system based on the [MOF-818@PMOF(Fe)] hybrid served as a mimic of the natural enzyme cascade. Initially, Cu-containing MOF-818 performs catechol oxidase activity, catalyzing the oxidation of 3,5-di-tert-butylcatechol (3,5-DTBC) and generating intermediate H_2_O_2_ in situ. Then, iron/porphyrin-containing MOF [PMOF(Fe)] utilized endogenous H_2_O_2_ to oxidize a chromogenic or chemiluminescent substrate, providing an analytical readout. Without the analyte, the aptamer suppresses the catalytic properties of the hybrid nanozyme. Binding with chlorpyrifos leads to the dissociation of the aptamer from the MOF-on-MOF hybrid, so the latter restores its catalytic activities. The proposed aptasensor provided sensitive and selective detection of pesticide in water samples.

Carbon-based nanomaterials (carbon nanotubes, graphene oxide, etc.) also possess peroxidase-like activity and could potentially be suitable for the generation of colorimetric signals. For instance, CD63-specific aptamer combined with graphitic carbon nitride nanosheets (g-C3N4 NSs) was used for exosome detection [64]. Without exosomes, DNA aptamer nonspecifically absorbs on the nanosheets, and due to additional negative charge, enhances their ability to catalyze TMB oxidation, providing a blue color of solution. Binding of the aptamer with CD63 on the exosome surface leads to a decrease in g-C3N4 NSs catalytic activity and a lower absorbance intensity. The proposed aptasensor discriminated exosomes produced by the MCF-7 cancer cell line and those from nontumorigenic cells. Moreover, the aptasensor was also suitable for exosome detection in serum samples from cancer patients and healthy donors.

A very similar SWCNT-based aptasensor for the detection of exosomes was developed by Y. Xia et al. [65]. DNA aptamer absorbed on SWCNTs enhances their catalytic activity, and in the presence of exosomes, aptamer dissociates from the nanotubes, resulting in a decrease in the colorimetric signal. The sensitivity of the proposed assay was comparable to the abovementioned g-C3N4 NSs-based aptasensor. The results of exosome detection in real serum samples were in agreement with those from commercial ELISA kit. 

A hybrid nanomaterial consisting of graphene oxide and Pt-Au NPs was also used for aptasensor design. The ATP-binding aptamer was cut into two fragments. The first fragment was conjugated with magnetic beads, and the second one was linked to GO/PtAuNPs. In the presence of ATP, two fragments of the aptamer form a specific complex with their target, and GO/PtAuNPs are thus linked with magnetic beads in one composite material. After magnetic separation, GO/PtAuNPs retain in the test tube and catalyze TMB oxidation, generating a blue color. Without ATP, GO/PtAuNPs are discarded after magnetic separation, and no color changes are registered. As PtAuNPs provided signal amplification, the LOD was 0.2 nM, much lower than that for other colorimetric biosensors.

Another hybrid nanomaterial made of reduced GO and ZnFe_2_O_4_ acted as a reporter in a sandwich system for *S. typhimurium* detection [66]. The first biotinylated aptamer was immobilized on the avidin-modified microplate for specific capture of the analyte. The second aptamer was conjugated to rGO/ZnFe_2_O_4_ and served as a reporter probe for analyte-aptamer complex visualization. The sensitivity of the proposed aptasensor was comparable to that for luminescent (LOD 11 CFU/mL) [67], and electrochemical (LOD 6 CFU/mL) [68] aptasensors for *S. typhimurium* detection.

### 2.3. Combination of Different HRP Mimics

HRP mimics of different types can also be combined to make an aptasensor. Such a combination strategy usually allows for significantly improved detection sensitivity thanks to the enhanced catalytic activity of composite peroxidase mimics and signal amplification. For example, Y. Zhang et al. used GQ-forming thrombin-specific aptamer binding hemin along with PtNPs for TMB oxidation [69]. Initially, aptamer is immobilized on magnetic beads via complementary interactions with DNA on their surface. Thrombin binding initiates a cascade of hybridization chain reactions, which give a concatemeric DNA with numerous G-quadruplexes. An incubation with hemin and Pt produced multiple DNAzymes and Pt nanoclusters that catalyze TMB oxidation. Without thrombin, Exo III nuclease selectively cleaves aptamer-DNA duplexes on magnetic beads and reduces background signal. The linear range of the system was 0.1–100 nM, with a LOD of 15 pM.

A combination of the hemin-DNAzyme complex with hybrid MOF/NPs was used for the sensitive detection of chloramphenicol [70]. A DNA hairpin containing an aptamer motif and a DNA fragment partially complementary to a DNAzyme is immobilized on the magnetic beads. A DNAzyme conjugated to PtNPs/MOF hybridizes with a DNA hairpin. After chloramphenicol addition, an aptamer binds with its target, displacing DNAzyme from the duplex. A released DNAzyme-hemin complex conjugated with PtNPs/MOF catalyzes TMB oxidation and generates a blue color. To enhance the sensitivity of detection, an analytical scheme was supplied by the circular strand-displacement polymerization reaction, so the LOD became as low as 0.03 pM. The proposed aptasensor was used for analyte detection in spiked milk samples, centrifuged and diluted about 500 times prior to the analysis. The recovery results were comparable to those for a commercial ELISA kit.

A. Rafati et al. [71] used a DNA-based hybrid nanomaterial for insulin detection. The GQ-forming insulin-binding aptamer was immobilized on the surface of a magnetic beads/DNA nanotube composite. After insulin binding, the aptamer folds into a hemin-binding GQ structure capable of TMB oxidation with the generation of blue color. In this system, DNA nanotubes amplify the colorimetric signal due to the local concentration of multiple aptamer molecules on one surface. The LOD was 2.6 pM, lower than that for the commercial ELISA kit (42 pM).

## 3. Conclusions

At the moment, several different types of non-protein peroxidase mimics have been developed to replace natural enzymes in biosensing systems. When an aptamer acts as an analyte-recognizing unit, hemin-binding peroxidase DNAzymes seem to be especially suitable as reporters due to the same chemical nature. In this case, the system is made of oligonucleotides, so it can be rationally designed and then fine-tuned in a number of ways by using auxiliary oligonucleotides or linker sequences. Nanozymes, such as metal nanoparticles, metal oxides, or carbon nanomaterials, possess their own advantages. In particular, gold nanoparticles represent a universal reporter for homogeneous assays. They acquire low peroxidase activity in an aptamer-bound state but restore the activity when the aptamer dissociates to bind its target. This strategy proved its feasibility for different aptamers and different targets, from small molecules to exosome vesicles. Otherwise, covalent conjugation with the aptamer provides the possibility of using nanozymes in heterogeneous sandwich assays. The catalytic activity of nanozymes can be regulated by varying their composition, thus offering a variety of ways for obtaining the desired sensitivity for a particular assay. A recently proposed novel class of nanozymes, metal-organic frameworks (MOFs), attracts special attention thanks to the highly controllable and reproducible characteristics. Some of them also possess fluorescent properties and are suitable for the design of dual-mode aptasensors. What is also important is that biosensors based on aptamers and peroxidase mimics have already shown their principal applicability for the analysis of biological, food, or environmental samples. 

By all means, specific characteristics of a desired analyte and their impact on aptasensor performance should be considered while developing aptasensors that recruit peroxidase mimics. When developing procedures for analysis, it is critical to take into account factors such as the pH and ionic strength of the analyte solution [10]. Moreover, aptamer selection could, if possible, be performed in experimental conditions that simulate the environment of their intended application.

All in all, the relative ease of manufacturing, high reproducibility of characteristics, and large possibilities for tuning sensitivity and specificity of detection according to the specified task make this type of biosensors a very promising alternative to protein-based assays, and we can surely expect an extension of their variety and field of application.

## Data Availability

Not applicable.

## Appendix A

**Table A1:** Aptamer-based detection systems recruiting non-protein peroxidase mimics.

Type of Peroxidase Mimic	Analyte	Substrate	LOD	Selectivity	Ref.
hemin-DNAzyme complex	ochratoxin A	TMB	2.5 nM	ochratoxin B warfarin	[19]
hemin-DNAzyme complex	AMP lysozyme	ABTS	50 μM for AMP 5 pM for lysozyme		[20]
hemin-DNAzyme complex	AMP lysozyme	ABTS	4 μM for AMP 0.1 pM for lysozyme		[21]
hemin-DNAzyme complex	ATP	ABTS	1 μM	TTP, CTP, GTP	[22]
hemin-DNAzyme complex	ATP cocaine	ABTS	5 μM for ATP 1 μM for cocaine		[23]
hemin-GQ aptamer	VEGF	luminol	18 nM		[24]
hemin-GQ aptamer	8-OHdG	ABTS	0.14 nM	8-OHdG analogs (dC, dA, dU, T, dG) and metal ions (Zn^2+^, Hg^2+^, Cu^2+^, Ca^2+^, Mn^2+^, Al^3+^, Na^+^)	[25]
hemin-DNAzyme complex	methamphetamine	ABTS	0.5 nM	15 general illicit drugs and metabolites: ketamine, norketamine, morphine, methadone, cocaine, mephedrone, cathinone, methcathinone, 3-trifluoromethylphenylpiperazine, 1-(3-tri-fluoromethylphenyl) piperazine, 3,4-methylenedioxy pyrovalerone, MDA, MDMA, EDDP, and mCPP	[26]
hemin-DNAzyme complex	СЕА	ABTS	5.5 pM	BSA, lysozyme, insulin, prostate-specific antigen	[27]
hemin-DNAzyme complex	СЕА	1,1′-oxalyldiimidazole	0.58 ng/mL	alpha-fetoprotein, prostate-specific antigen	[28]
hemin-DNAzyme complex	VEGF	ABTS	1.7 pM		[29]
hemin-DNAzyme complex	*S. aureus*	luminol	5 CFU/mL	*S. epidermidis*, *S. warneri*, *P. aeruginosa*; *E. coli*; *C. Perfringens*, and inactivated *S. aureus*	[30]
hemin-DNAzyme complex	ATP	ABTS			[32]
hemin-DNAzyme complex	cocaine	ABTS	1 μM	chlorpromazine, diphenhydramine, promazine, scopolamine, caffeine, levamisole, lidocaine, and sucrose. minimal cross-reactivity to caffeine, chlorpromazine, promazine, and levamisole	[33]
hemin-DNAzyme complex	MUC-1 (exosomes)	ABTS	3.94 × 10^5^ particles/mL	exosomes generated by normal liver cell line L-02	[34]
hemin-DNAzyme complex	quinclorac	TMB	7.1 ng/mL	other quinolines and commonly used pesticides	[35]
hemin-DNAzyme complex	MUC1	ABTS	5 nM	BSA, thrombin, lysozyme, IgG	[36]
hemin-DNAzyme complex	ATP	TMB	2.4 nM	CTP, GTP and UTP	[31]
hemin-DNAzyme complex	Hg^2+^ ions thrombin sulfadimethoxine cocaine 17β- estradiol	ABTS	4 μM for thrombine 0.5 μM for sulfadimethoxine		[37]
hemin-DNAzyme complex	ochratoxin A	TMB	10 nM	other mycotoxins: aflatoxin B1, zearalenone, and ochratoxin B	[38]
nanozyme: PtNPs	thrombin	TMB	0.4 μM		[39]
nanozyme: Ag/Pt nanoclusters	thrombin	TMB	2.6 nM	HSA, lysozyme, IgG, bovine thrombin	[40]
nanozyme: AuNPs	kanamycin	TMB	1.49 nM	penicillin, ampicillin, and streptomycin	[41]
nanozyme: AuNPs	acetamiprid	TMB	1.8 ppm	agritone, imidacloprid, and endothal	[42]
nanozyme: AuNPs	sulfadimethoxine	TMB	10 ng/mL	kanamycin, chloramphenicol, oxytetracycline hydrochloride	[43]
nanozyme: AuNPs	murine norovirus	TMB	30 particles/mL	*S. aureus*, *E. coli*, *E. coli* bacteriophage MS2	[44]
nanozyme: AuNPs	*P. aeruginosa*	TMB		*V. cholerae*, *L. monocytogens*, *S. aureus*	[45]
nanozyme: AuNPs	zearalenone	TMB	10 ng/mL	aflatoxin B1, ochratoxin B, and metal ions (Ca^2+^, Na^+^, Mg^2+^, Zn^2+^)	[46]
nanozyme: AuNPs	streptomycin	ABTS	86 nM	tetracycline, oxytetracycline, carbamazepine, penicillin, amoxicillin, and diclofenac	[47]
nanozyme: AuNPs	CD30+ small extracellular vesicles	TMB	10^2^–10^9^ vesicles/mL		[48]
nanozyme: AuNPs	malachite green	TMB	1.8 nM	sulfaguanidine, sulfanilamide, oxytetracycline hydrochloride, chloramphenicol	[49]
nanozyme: AuNPs	sIL-2Ra	oPD	1 pM	BSA, IL-5Ra, IL-13Ra2, IL-17Ra, CD166	[50]
nanozyme: AuNPs	C-reactive protein	TMB	0.07 pM		[51]
nanozyme: AuNPs	retinol-binding protein 4	luminol	50 fM		[52]
nanozyme: AgNPs (tyrosine-capped)	chlorpyrifos	TMB	11.3 ppm	diazinon, dichlorvos, phorate, monocrotophos, methamidophos, azamethiphos, aldicarb, clothianidin, captan, thiamethoxam, mancozeb	[53]
nanozyme: Fe_3_O_4_	*S. typhimurium*	TMB	7.5 × 10^5^ CFU/mL		[54]
nanozyme: Au/Pd	*C. jejuni*	TMB	10 cells	*H. pylori*, *E. coli*	[55]
nanozyme: core-shell gold nanorods	glucose insulin	TMB	7.5 μM for glucose 0.2 pM for insulin		[56]
nanozyme: Au nanoclusters conjugated to BSA	*S. typhimurium*	TMB	1 CFU/mL	*E. coli* O157:H7, *S. aureus*, *P. aeruginosa*	[57]
nanozyme: Fe-MOF	thrombin	TMB	0.8 nM	BSA, lysozyme, IgG, amino acids	[58]
nanozyme: Cu-MOF	C-reactive protein	TMB	240 pg/mL	glucose, glutathione, ascorbic acid, iron, creatinine, albumin, calcium	[59]
nanozyme: Cu-MOF	thrombin	TMB	360 pM	glucose, glutathione, ascorbic acid, iron, creatinine, albumin, C-reactive protein, calcium	[60]
nanozyme: MOF	chlorpyrifos	TMB	4.4 ng/mL	atrazine, carbaryl, diazinon, malathion, bisphenol A, imidacloprid	[61]
nanozyme: MOG + PtNPs	fumonisin B1	TMB	2.7 pg/mL	ochratoxin A, ochratoxin B, aflatoxin B1, aflatoxin B2, zearalenone	[62]
nanozyme: MOF-on-MOF	chlorpyrifos	TMB luminol	5.3 ng/mL	Na^+^, K^+^, Mg^2+^, Zn^2+^, Cl^−^, NO^3−^, SO4^2−^, Ac^−^, glucose, urea, citric acid, glyphosate, trichlorfon	[63]
nanozyme: g-C3N4 NSs	CD63+ exosomes	TMB	13.52 × 10^5^ particles/μL	exosomes generated by nontumorigenic cell line	[64]
nanozyme: SWCNTs	CD63+ exosomes	TMB	5.2 × 10^5^ particles/μL	exosomes generated by nontumorigenic cell line	[65]
nanozyme: ZnFe_2_O_4_/rGO	*S. typhimurium*	TMB	11 CFU/mL	*S. aureus*, *E. coli*, *L. monocytogenes*, *V. parahaemolyticus*	[66]
hybrid: DNA nanotube/magnetic beads/ hemin-DNAzyme	insulin	TMB	0.39 µIU/mL		[71]
hybrid: hemin-DNAzyme/PtNPs	thrombin	TMB	15 pM	hemoglobin, lysozyme, BSA	[69]
hybrid: DNAzyme + MOF + PtNPs	chloramphenicol	TMB	0.03 pM	kanamycin, streptomycin, oxytetracycline, gentamicin sulfate, chlortetracycline, Zn^2+^, Ca^2+^, Mg^2+^, casein, globulin, albumin	[70]
